# Optimization of the Real-Time Quaking-Induced Conversion Assay for Prion Disease Diagnosis

**DOI:** 10.3389/fbioe.2020.586890

**Published:** 2020-11-19

**Authors:** Inga Zerr, Maria Cramm, Susana Margarida da Silva Correia, Saima Zafar, Anna Villar-Piqué, Franc Llorens, Matthias Schmitz

**Affiliations:** ^1^Department of Neurology, German Center for Neurodegenerative Diseases (DZNE), University Medical Center Göttingen, Göttingen, Germany; ^2^Biomedical Engineering and Sciences Department, School of Mechanical and Manufacturing Engineering, National University of Sciences and Technology, Islamabad, Pakistan; ^3^Bellvitge Biomedical Research Institute, Hospitalet de Llobregat, Barcelona, Spain; ^4^Center for Networked Biomedical Research on Neurodegenerative Diseases, Institute of Health Carlos III, Hospitalet de Llobregat, Barcelona, Spain

**Keywords:** cerebrospinal fluid, Creutzfeldt-Jakob disease, diagnostic test, prion protein, real-time quaking-induced conversion

## Abstract

The real-time quaking-induced conversion (RT-QuIC) assay is a highly reproducible and robust methodology exhibiting an excellent pre-mortem diagnostic accuracy for prion diseases. However, the protocols might be time-consuming and improvement of the detection technology is needed. In the present study, we investigated the influence of a pre-analytical cerebrospinal fluid (CSF) treatment with proteinase K (PK) on the kinetic of the RT-QuIC signal response. For this purpose, we added PK at different concentrations in RT-QuIC reactions seeded with Creutzfeldt–Jakob disease (sCJD) CSF. We observed that a mild pre-analytical PK treatment of CSF samples resulted in an increased seeding efficiency of the RT-QuIC reaction. Quantitative seeding parameters, such as a higher area under the curve (AUC) value or a shorter lag phase indicated a higher conversion efficiency after treatment. The diagnostic accuracy resulting from 2 μg/ml PK treatment was analyzed in a retrospective study, where we obtained a sensitivity of 89%. Additionally, we analyzed the agreement with the previously established standard RT-QuIC protocol without PK treatment in a prospective study. Here, we found an overall agreement of 94% to 96%. A Cohen’s kappa of 0.9036 (95% CI: 0.8114–0.9958) indicates an almost perfect agreement between both protocols. In conclusion, the outcome of our study can be used for a further optimization of the RT-QuIC assay in particular for a reduction of the testing time.

## Introduction

The pre-mortal diagnostic criteria of Creutzfeldt–Jakob disease (sCJD) include clinical examinations combined with magnetic resonance imaging (MRI) of the brain, electroencephalography (EEG), and laboratory analysis of cerebrospinal fluid (CSF; WHO, 1998, 2003; Zerr et al., 2009). Surrogate biomarkers such as 14-3-3, tau, alpha synuclein, etc. were applied for pre-mortem diagnosis (Zerr et al., 1998; Llorens et al., 2016, 2017; Schmitz et al., 2016c,d, 2019). A major innovation in prion disease diagnostic was the development of *in vitro* protein misfolding cyclic amplification (PMCA) assays. These include the PMCA (Saborio et al., 2001), the amyloid seeding assay (ASTA; Colby et al., 2007), the quaking-induced conversion (QuIC; Atarashi et al., 2008), and the real-time quaking-induced conversion (RT-QuIC; Atarashi et al., 2011; McGuire et al., 2012; Orru et al., 2014; Schmitz et al., 2016a,b). Atarashi et al. (2011) firstly used the RT-QuIC to amplify minuscule amounts of a PrP^Sc^ seed in CSF from patients with prion disease. Afterward, a high specificity of approximately 100%, robustness, and high reproducibility of the RT-QuIC had been demonstrated by several groups (McGuire et al., 2012, 2016; Sano et al., 2013; Cramm et al., 2015, 2016). The sensitivity of the RT-QuIC depends on the kind of prion disease (Sano et al., 2013; Cramm et al., 2015); for sCJD, it varies between 80% and 95%. Other confounding factors are the composition of the study cohort or the type of recombinant PrP^C^ (hamster, hamster–sheep, human, bank vole, etc.; Orru et al., 2015; Candelise et al., 2017).

Recently, a suggestion has been made to amend the criteria by adding the PrP^Sc^ detection by RT-QuIC (Hermann et al., 2018). Therefore, a further optimization of the RT-QuIC by reducing the average testing time of 80 h would be beneficial.

In the present study, we analyzed the influence of a mild pre-analytical proteinase K (PK) treatment of CSF samples (sCJD and controls) on the PrP conversion efficiency in the RT-QuIC as defined by quantitative seeding parameters, such as the lag phase or the area under the curve (AUC). After selection of a PK concentration, we performed a retrospective study to define the sensitivity and a prospective study to compare the accordance of the RT-QuIC with and without pre-analytical PK treatment.

## Materials and Methods

### Ethics Statement

The studies involving human CSF samples were reviewed and approved by the local Ethics Committee of the University Medicine Göttingen, Von Siebold-Str. 37075 Göttingen, No. 24/8/12. All samples were analyzed blinded for at least personal data.

### Patients

We performed a retrospective study including 65 sCJD patients [35 patients with codon 129 homozygous methionine genotype (MM), 15 heterozygous MV, and 15 VV cases] as well as 20 control subjects without prion disease. All sCJD cases were diagnosed as probable cases according to diagnostic consensus criteria; 20 were confirmed by autopsy (WHO, 1998, 2003; Zerr et al., 2009). From them, we used four sCJD MM cases for studying the influence of PK incubation and deactivation steps on RT-QuIC signal response.

The control group included patients either healthy or with clinically defined alternative diagnosis such as Alzheimer’s disease, alpha synucleinopathies, or psychiatric disorders (psychosis, bipolar disorder, and schizophrenia). Additionally, we included a prospective cohort that consisted of 35 RT-QuIC positive cases (suspected sCJD cases) and 51 cases exhibiting a negative RT-QuIC seeding response (suspected controls). These samples were collected during diagnostic routine without any further characterization.

### RT-QuIC Analysis

We conducted the RT-QuIC analysis according to an established protocol (Schmitz et al., 2016b, 2018). Each sample was run in triplicates. Briefly, 85 μl of RT-QuIC buffer [162 mM phosphate buffer (pH 6.9), 170 mM sodium chloride, 1 mM ethylenediaminetetraacetic acid (EDTA), 10 μM Thioflavin-T (Th-T), and 0.1 mg/ml recPrP] was seeded with 15 μl of CSF (total volume of 100 μl). For RT-QuIC analysis, we used the BMG OPTIMA FLUOStar plate reader. Reactions were run at 42°C for 80 h with intermittent shaking cycles [1-min double orbital shaking at the highest speed (600 rpm)] followed by 1-min incubation. Every 30 min, fluorescence was measured at 450-nm excitation and 480-nm emission.

### Pre-analytical PK Treatment

Cerebrospinal fluid samples were subjected to a mild pre-analytical treatment with PK in a range of 0–6 μg/ml. After adding PK, samples were incubated for 30 min at 37°C. Subsequently, PK was deactivated by heating at 65°C for 30 min.

### Coomassie Brilliant Blue Staining

Acrylamide gels (12%) were fixed in a solution containing 50% methanol and 10% glacial acetic acid for 1 h. After 1 h, solution was exchanged. Staining with 1% Coomassie brilliant blue R-250, 50% methanol, and 10% glacial acetic occurred for 20 min with gentle agitation. Destaining in 40% methanol and 10% glacial acetic acid solution was carried out until the gel background was fully transparent destained. Finally, gels were stored at RT in 5% glacial acetic acid solution.

### Statistical Analysis

Agreement between the standard and the modified protocol was measured with the Cohen’s kappa statistics, available in the vcd R package (Meyer et al., 2020). Then, measures of seeding efficiency were calculated for each individual sample defined by relative area under the curve (rel. AUC) and lag phase (time to 50% signal increase). Multiple comparisons data were analyzed by one-way ANOVA followed by Dunn’s *Post hoc* test. Two groups (normally distributed) were compared using the Student’s *t* test, while for non-normally distributed values, we considered the Mann–Whitney *U* test as appropriate. All analyses were conducted by using GraphPad Prism 8.

## Results

### Influence of Serial Pre-analytical PK Treatments on the RT-QuIC Seeding Response

We performed a mild pre-analytical treatment of CSF samples with PK (2 μg/ml). After adding PK, samples were incubated for 30 min at 37°C. Subsequently, PK was deactivated by heating at 65°C for 30 min.

**FIGURE 1:**
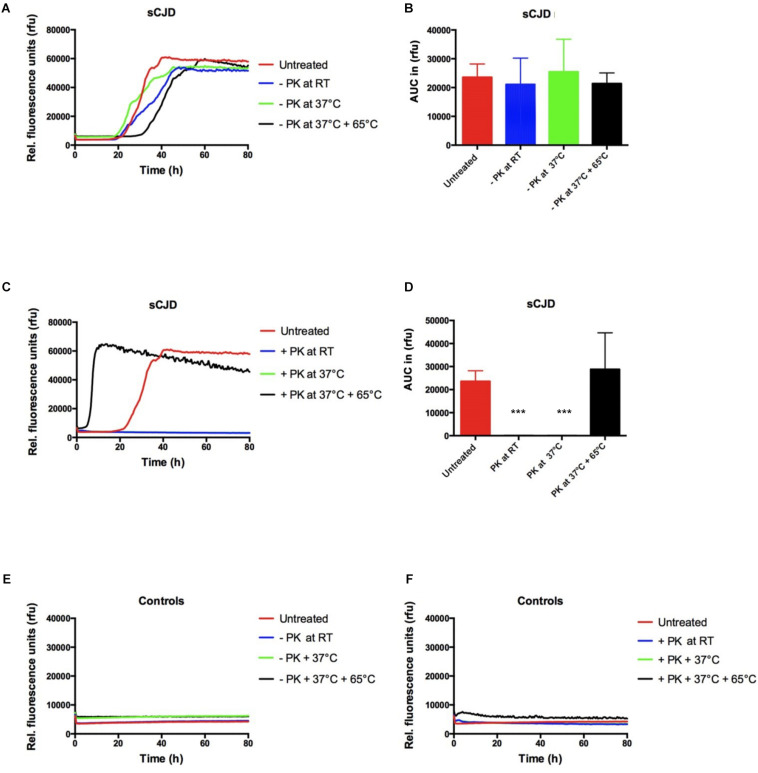
Influence of PK incubation and deactivation steps on the RT-QuIC signal response. **(A,B)** All incubation and deactivation steps without PK (addition of water instead of PK) had no effect on the seeding kinetic and the AUC values in sCJD (MM; *n* = 4) seeded RT-QuIC reactions. **(C,D)** PK treatment (2 mg/ml) requires a deactivation step (30 min at 65°C); otherwise, no seeding reaction is observable when incubating with PK at room temperature (RT) or at 37°C. **(E,F)** In control-seeded reactions (*n* = 4), neither the incubation of 30 min at RT or at 37°C nor the deactivation for 30 min at 65°C exhibited a significant influence on the signal response independently from PK treatment. *p* values: <0.001 as extremely significant (***).

The incubation step for 30 min at RT or at 37°C alone or in combination with the incubation of 30 min at 65°C (without PK) exhibited a non-significant effect on signal response compared to untreated reactions (Figures 1A,B).

In PK-treated reactions, the deactivation step is required; otherwise, all sCJD (MM) seeded reactions showed a negative seeding response comparable to controls (Figures 1C,D). In control seeded reactions, none of the conditions had changed the seeding kinetics (Figures 1E,F).

**FIGURE 2:**
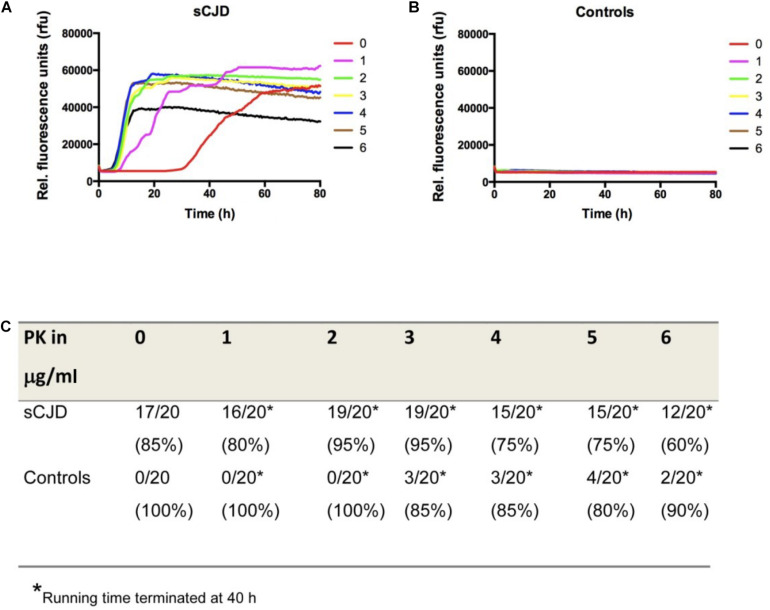
Influence of different amounts of PK on the RT-QuIC seeding response. **(A,B)** RT-QuIC reactions seeded either with sCJD MM or with control CSF were treated with different amounts of PK (0–6 μg/ml). The kinetics of positive RT-QuIC signaling responses indicated that treatment with PK influences the conversion efficiency of PrP in comparison to untreated reactions. **(C)** Accuracy of the RT-QuIC before and after PK treatment suggested a pre-treatment with 2 μg/ml PK as most suitable for a reliable diagnostic. For simplification, we displayed the means of all positive sCJD and negative control seeded reactions per group at each point in time and not the false-positive or -negative reactions.

Subsequently, 20 well-characterized CSF samples from sCJD (MM) patients and 20 controls without prion disease were treated with different PK concentrations (0–6 μg/ml). After PK treatment, samples were subjected to RT-QuIC analysis using the standard protocol (Cramm et al., 2016; Schmitz et al., 2016b, 2018), which included recombinant hamster–sheep as substrate. The signal-kinetic in the RT-QuIC reaction was measured in dependency from the PK concentration (0–6 μg/ml). It indicated a significant increase of the conversion efficiency of reactions, seeded with sCJD CSF after PK treatment (Figures 2A,B). To define the optimal assay conditions for the sCJD diagnosis, we validated the correctly classified sCJD and control cases. Interestingly, the pre-analytical PK treatment of CSF samples in a concentration of 2 μg/ml revealed the best diagnostic accuracy; 19/20 sCJD cases and 0/20 control cases showed over a duration of 40 h testing time a positive RT-QuIC signal response (Figure 2C). The use of higher PK amounts and longer testing times may result in false-positive RT-QuIC reactions (Figure 2C).

### Pre-analytical PK Treatment (2 μg/ml) Significantly Reduced the Lag Phase and the Testing Time of the RT-QuIC

The impact of a pre-analytical CSF treatment with 2 μg/ml PK on the total protein amount and the quantitative seeding parameters (such as lag phase and AUC) was now analyzed more in detail.

At first, we compared the total protein amount in sCJD MM CSF samples before and after PK treatment (2 μg/ml) by gel electrophoresis followed by *Coomassie Brilliant Blue* staining. Quantification of band intensities occurred by *Image Lab* software. Interestingly, we observed total protein amounts up to 50% decreased after PK treatment (Supplementary Figures 1A,B).

**FIGURE 3:**
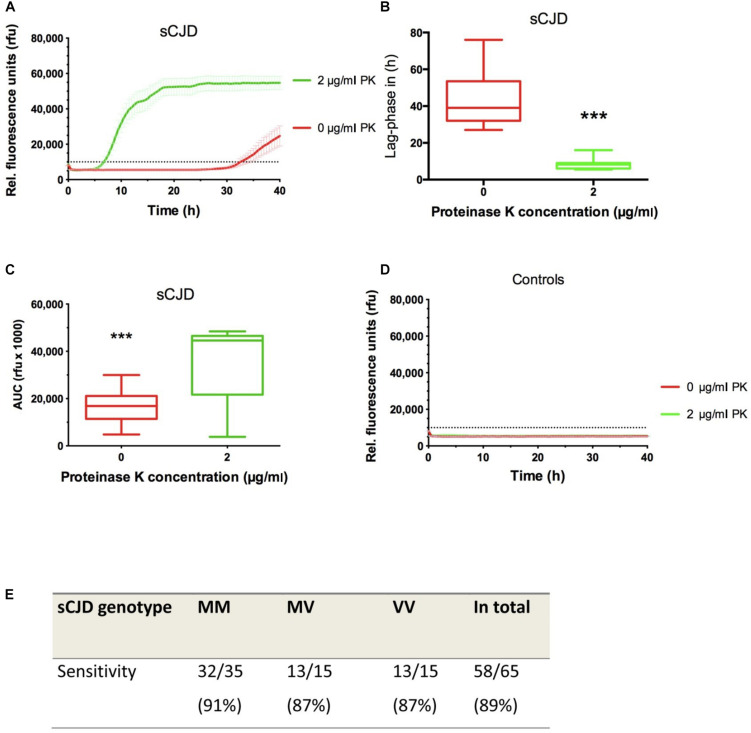
Optimization of the CSF RT-QuIC. **(A)** RT-QuIC reactions terminated at 40 h and seeded with sCJD MM CSF (*n* = 20) were treated with 2 μg/ml of PK, which increased the conversion efficiency of PrP significantly as indicated by a shorter lag phase **(B)** and a higher relative area under the curve (AUC) value **(C)**. Non-prion disease controls (*n* = 20) remained negative **(D)**. **(E)** In a retrospective study, we analyzed the sensitivity of the RT-QuIC after PK (2 μg/ml) treatment in different sCJD codon 129 MV genotypes showing an average sensitivity of 89% for all sCJD codon 129 MV genotypes. RT-QuIC responses were measured in rfu over a period of 40 h. Displayed are means ± SEM (standard error of the mean) at each point in time. *p* values: <0.001 as extremely significant (***).

Next, we calculated quantitative seeding parameters of untreated in comparison to PK (2 μg/ml)-treated RT-QuIC reactions seeded with sCJD MM CSF. The obtained data indicated that RT-QuIC reactions pre-analytically exposed to PK revealed a significantly shorter lag phase, which decreased from more than 30 h (untreated) to 6–9 h on average as well as an increased AUC value after PK treatment (Figures 3A–C), while controls remained on a baseline level (Figure 3D).

### Determination of the Sensitivity After PK Treatment in a Retrospective Study

The sensitivity of RT-QuIC after pre-analytical treatment with PK was analyzed in a retrospective study. The cohort consisted of 65 sCJD patients (35 MM, 15 MV, and 15 VV). After pre-analytical exposure to 2 μg/ml PK, samples were analyzed via RT-QuIC for 40 h. The sensitivities varied between different sCJD codon 129 MV genotypes between 87% and 91%, while the average sensitivity for all sCJD genotype was 89% (Figure 3E).

### Examination of the Agreement Between Optimized and Standard RT-QuIC

To determine the analytical agreement of the optimized RT-QuIC (with PK 2 μg/ml) and the standard RT-QuIC, a prospective study on a patient cohort consisting of 35 samples with a positive RT-QuIC response as well as 51 samples exhibiting a negative standard RT-QuIC response was performed. PK-treated and untreated samples were analyzed in parallel on the same plate. Interestingly, we obtained an agreement of 94% for the RT-QuIC positively classified cases and 96% for all CSF samples exhibiting a negative RT-QuIC signal response (Table 1). In addition, we calculated the Cohen’s kappa value to determine the agreement between the standard and the optimized RT-QuIC protocol. Interestingly, we obtained a value of 0.9036 (95% CI: 0.8114–0.9958), which indicates an almost perfect agreement between both protocols.

**TABLE 1:** In a prospective study, we analyzed the agreement of untreated (standard) and PK (2 μg/ml)-treated (optimized) RT-QuIC reactions.

Prospective study	Untreated	2 μg/ml PK treated	Agreement in %
RT-QuIC positive reactions	35	33	94%
RT-QuIC negative reactions	51	49	96%

*We selected CSF samples with positive or negative signal response in the standard RT-QuIC and analyzed them in parallel in the optimized RT-QuIC.*

## Discussion

Until now, several *in vitro* protein misfolding amplification systems, such as the RT-QuIC, have been established providing relevant improvements in prion disease diagnostics and prion research (Atarashi et al., 2011; McGuire et al., 2012; Sano et al., 2013; Cramm et al., 2015, 2016). The standard diagnostic RT-QuIC protocol for the analysis of sCJD CSF (with recombinant hamster–sheep PrP^C^ as a substrate) exhibited a total assay duration for testing of 80 h (Cramm et al., 2015, 2016; Schmitz et al., 2016a,b, 2018). The lag phase of a positive RT-QuIC reaction lasts 30–40 h in average with accuracy between 80 and 85% and a specificity of almost 100% (Cramm et al., 2016). In the present study, we intend to modify the RT-QuIC standard protocol to reduce the lag phase and the testing time of the RT-QuIC.

### Optimization of sCJD RT-QuIC by a Pre-analytical PK Treatment

In an initial study, we included a mild PK treatment of CSF in the pre-analytical protocol. Afterward, CSF samples were subjected to the standard RT-QuIC analysis (Cramm et al., 2016; Schmitz et al., 2016b, 2018). To elucidate the optimal amount of PK, we performed a serial dilution from 0 to 6 μg/ml of PK. All PK concentrations ≥2 μg/ml reduced the lag phase significantly. However, the diagnostic accuracy of the optimized RT-QuIC depended on the amount of PK. The usage of ≥3 μg/ml of PK resulted in a significant decrease of the specificity by creating false-positive RT-QuIC signals, as revealed by control RT-QuIC reactions (CSF without prion disease). A 2 μg/ml PK treatment provided the best diagnostic accuracy and allowed a reduction of the testing time from 80 to 40 h. Under these conditions, the PrP conversion process is accelerated, as indicated by a reduced lag phase and an increase of AUC values compared to the standard protocol without PK treatment. A potential reason may be that PK treatment resulted in a decrease of total protein amounts of more than 50%. Lower protein levels may affect the PrP^C^–PrP^Res^ interaction in the RT-QuIC, which most likely promotes the conversion efficiency. Alternatively, it could be that PK partially digests external parts of PrP^Res^ species. This way, inner beta strands may become more exposed, displaying a higher seeding capacity.

To examine the application to all sCJD codon 129 MV genotypes and define the diagnostic sensitivity, a retrospective study was performed by analyzing 65 confirmed sCJD cases. Here, we obtained an average sensitivity for all genotypes of 89%. This is an increase compared to the standard RT-QuIC protocol (Cramm et al., 2016; Schmitz et al., 2016b, 2018), whereas the number of samples was lower.

In addition, we investigated the agreement of the new protocol (with PK) to the previous standard protocol in a prospective study. The accordance between both protocols varied between 94 and 96% with a Cohen’s kappa of 0.9036 (95% CI: 0.8114–0.9958), which indicates an almost perfect agreement between both protocols. For four CSF samples, where we do not have any follow-up information, both protocols disagreed. One explanation may be an increase of sensitivity of the RT-QuIC after PK treatment. However, to finally elucidate this issue, a retrospective study on a higher number of well-characterized samples (e.g., >200 sCJD samples, consisting of typical and atypical sCJD cases as well as >100 neurological controls) is recommended.

Besides the standard or first generations of the RT-QuIC, a few studies successfully applied a so termed second generation of the RT-QuIC assay. In the second generation of the RT-QuIC, a truncated Syrian hamster recombinant PrP substrate (amino acids 90–231) and some modifications [incubations at 55°C instead of 42°C, and the addition of 0.002% sodium dodecyl sulfate (SDS)] of the standard RT-QuIC protocol have been used (Foutz et al., 2017; Franceschini et al., 2017; Groveman et al., 2017). In contrast to the full-length recombinant PrP substrate, the use of truncated recombinant PrP as a substrate contributed to an acceleration of the RT-QuIC seeding response and an increase of the conversion efficiency. The lag phase (less than 20 h) and the detection times (2-day reduction in average detection time) were significantly reduced in the second generation of the RT-QuIC (Foutz et al., 2017; Franceschini et al., 2017; Groveman et al., 2017), which is comparable to the optimized protocol with PK treatment. Moreover, the sensitivity of the RT-QuIC could be increased (up 94%, 113 CJD cases were tested) without loss of specificity (100%; Foutz et al., 2017; Franceschini et al., 2017; Groveman et al., 2017; Thompson and Mead, 2019).

In summary, our study provides an alternative to the originally as second generation described RT-QuIC for a further optimization of this test. The pre-analytical treatment of CSF with PK helps to reduce the testing time by increasing the conversion efficiency indicated by a shorter lag phase, higher AUC values, and an increase of the sensitivity.

## Data Availability Statement

All datasets presented in this study are included in the article/Supplementary Material.

## Ethics Statement

The studies involving human participants were reviewed and approved by local ethics committee in Göttingen (No. 24/8/12). The patients/participants provided their written informed consent to participate in this study.

## Author Contributions

IZ and MS conceived the study. MC and SC performed experiments. IZ, MC, FL, and MS analyzed and interpreted data. IZ and MS drafted the manuscript. SZ, AV-P, and FL critically revised the manuscript. All authors read and approved the final manuscript version.

## Conflict of Interest

The authors declare that the research was conducted in the absence of any commercial or financial relationships that could be construed as a potential conflict of interest.

## Supplementary Material

Supplementary Figure 1 Decrease of total protein amounts after treatment with PK 2 μg/ml **(A)** CSF samples from sCJD MM patients either treated with PK or untreated were stained with Coomassie brilliant blue. **(B)** Densitometric analysis via Image Lab indicated a decrease of total protein amount after treatment with PK.
