# Supplementary figures and images for: Optimization of the Real-Time Quaking-Induced Conversion Assay for Prion Disease Diagnosis
Source: Front Bioeng Biotechnol. 2020 Nov 19;8:586890. doi: 10.3389/fbioe.2020.586890 (PMC7710546; doi:10.3389/fbioe.2020.586890)

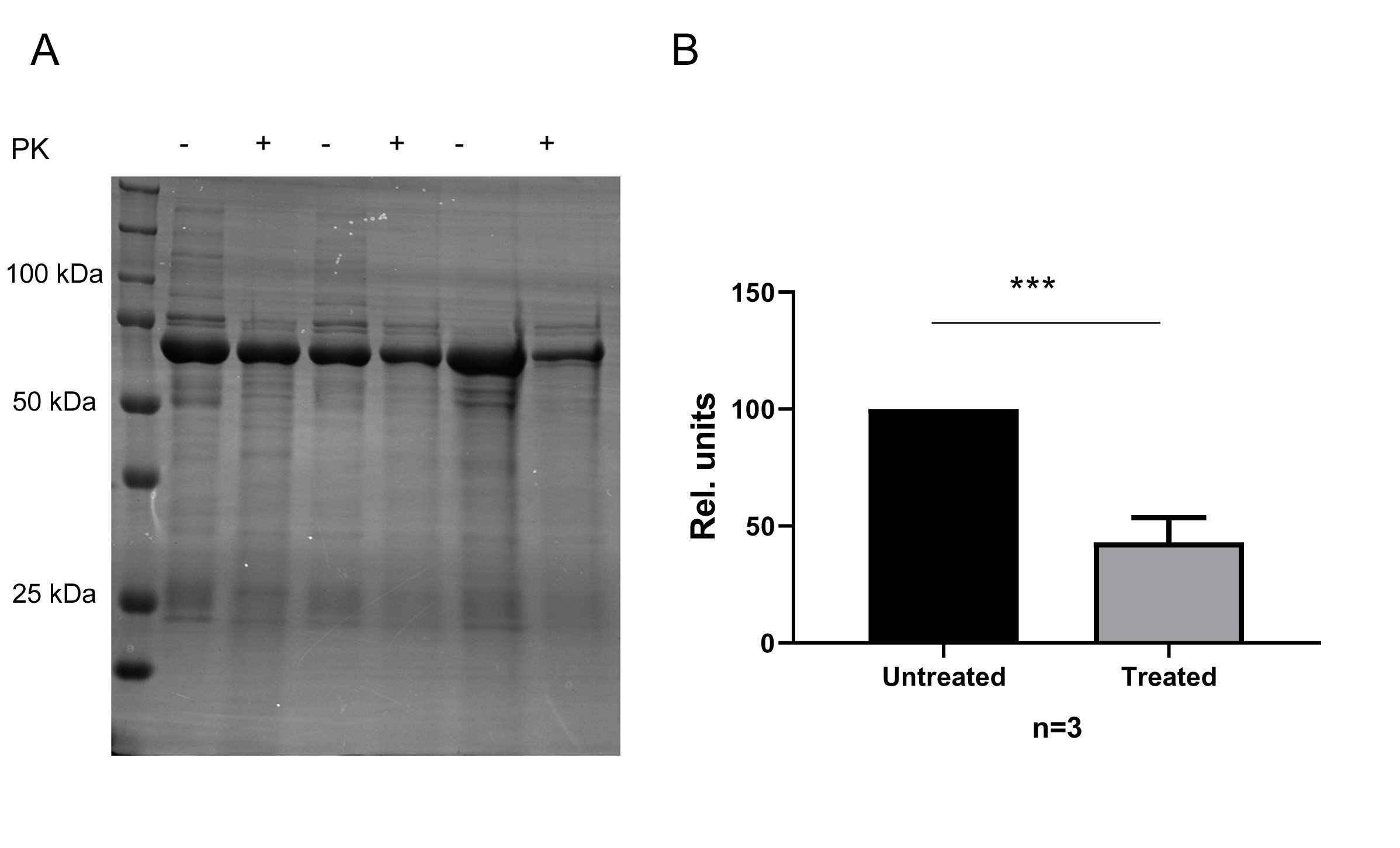

Supplement: Supplementary Figure 1 — Decrease of total protein amounts after treatment with PK 2 μg/ml (A) CSF samples from sCJD MM patients either treated with PK or untreated were stained with Coomassie brilliant blue. (B) Densitometric analysis via Image Lab indicated a decrease of total protein amount after treatment with PK. [file Image_1.TIF]
